# Search and Study of Marked Code Structures for a Spatially Distributed System of Small-Sized Airborne Radars

**DOI:** 10.3390/s23156835

**Published:** 2023-07-31

**Authors:** Vadim A. Nenashev, Sergey A. Nenashev

**Affiliations:** Saint-Petersburg State University of Aerospace Instrumentation, 67, Bolshaya Morskaia Str., 190000 Saint-Petersburg, Russia; nenashev_sergey178@mail.ru

**Keywords:** marked code structures, Barker codes, M-sequences, signal modulation, correlation characteristics, system of code structures, spatially distributed system, small-sized airborne radar, integrated processing of echo signals

## Abstract

When forming the radar situation of a terrain, in order to increase its information content and to extract useful information, multi-position spatially distributed systems for integrating multi-angle radar data established by small-sized UAV-based airborne radars are used. In this case, each radar station belonging to a multi-position system as a probing signal must have its own unique marked signal. Such a setup will allow the signals reflected from ground objects and zones to be “attached” to specific receiving-transmitting positions of the multi-position system. This requirement results from the fact that each transceiver position emits one probing signal, and then receives all the echo signals reflected from the underlying surface and previously emitted by other radar devices of the multi-position system. Such a setup of multi-position systems requires the researcher to look for and investigate specialized systems of marked code structures used to modulate the probing signals in order to identify them in a joint radar channel. Thus, the problem at hand is how to look for and investigate novel marked code structures used to generate a system of probing signals, the use of which will allow it to be “attached” to a specific receiving-transmitting position of a multi-position onboard system and to identify them in a joint radar channel in the course of the remote sensing of the underlying surface. The purpose of this work is to conduct a study on the subject of the squeak and choice of a system of code structures that have a low level of side lobes of autocorrelation functions and uniformly distributed values of the levels of the cross-correlation function. To achieve this goal, the relevance of the study is substantiated in the introduction. The second section analyzes the level of side lobes for classical and modified Barker codes with an asymmetric alphabet. On the basis of this analysis, it was concluded that it is expedient to apply this approach for codes longer than Barker codes. Therefore, in the third section, the features of the generation of M-sequences are considered. The fourth section presents a technique for searching for new marked code structures, taking into account their mutual correlation properties for modifying M-sequences in order to implement multi-positional systems. The fifth section presents computer experiments on the search for marked code structures based on the modifications of M-sequences and presents the numerical characteristics of the correlation properties of the considered marked codes. And finally, in the sixth section, the final conclusions of the study are presented and recommendations are given for their further practical application. The practical significance of this study lies in the fact that the proposed new marked code structures are necessary for the synthesis of probing signals in the implementation of spatially distributed systems that function for the high-probability detection and high-precision determination of the coordinates of physical objects and are also necessary for the formation of radar images in a multi-position mode.

## 1. Introduction

One of the most important tasks in the design of airborne radar systems is to increase the resolution of the onboard small-sized radio-electronic equipment. This factor is of primary importance because of the detailed composition of the radar images of the earth’s surface using an airborne radar (AR).

High resolution in the range coordinate is achieved through the use of integrated probing signals, which are determined by a wide spectrum B of the emitted pulse. To determine the range coordinate with high resolution, the width of the spectrum of the pulse signal is chosen to be sufficiently large [1,2,3,4,5,6,7,8,9].

One of the promising areas for the development of airborne monitoring systems, which enables these systems to be extended with new properties, is the transition to a multi-position (containing several transmitting and receiving positions spaced apart in space) AR [10,11,12,13,14,15,16]. Along with the traditional approach to designing these systems, the possibilities of implementing a spatially distributed (SD) AR using multiple access technology have recently been considered [17,18].

The use of this technology ensures the high performance of the AR while remaining within the framework of active radar methods, for example, to achieve noise immunity, high resolution, detection characteristics, accurate determination of the object coordinates, etc. [11,12,14,15,19]. One of the distinguishing features of such an AR is the use of probing signals in combination. The most comprehensive high requirements for multi-position SD are met when using broadband signals.

When constructing such SD, it is necessary to choose a system of probing signals that will provide both high signal compression characteristics and a low degree of mutual influence of signals in a joint radar channel for integrated data processing.

In this regard, in the development of multi-position radars, it is strongly required to use multiple access technology, which requires the search and selection of specific probing signal systems. The process of searching and selecting a system of probing signals for multi-position assemblies is complicated by the following fact: it is required to organize their functioning under conditions of mutual influences. In this case, it is necessary not only to provide high compression ratios based on the autocorrelation characteristics of the selected ensemble of echo signals, but also to estimate the degree of mutual influence of these signals on each other, which is determined from their characteristics of the cross-correlation function (CCF). To do this, we consider code structures for broadband signal modulation used in radar systems for the possible unambiguous identification of such signals when emitted from each position of the AR in a multi-position SD.

Thus, when designing such SD, it is required to choose a system of code structures for the modulation of the probing signals that will provide both high compression characteristics of these signals and little mutual influence of these signals in a joint radar channel being used for integrated data processing. The aspects described above define the problem of finding such a system of code structures for the modulation of wideband signals, regarding the requirements for which, the following prerequisites should be met. On one hand, the CCF of any pair from the group of code sequences of the same length should be uniformly distributed, and on the other hand, a low level of the side lobes (SL) autocorrelation function (ACF) should be achieved.

Further, we analyze the well-known code structures to evaluate their ACF and CCF, taking into account the search for their modifications in order to mark them and use them in a joint channel for the integrated processing of radar data.

## 2. Analysis of the Level of Side Lobes of Modified Barker Codes with Asymmetric Alphabet

At present, aiming to increase the noise immunity of the channels of radar on-board systems, methods of compressing complex signals, in particular signals with phase modulation (PM) and amplitude modulation (AM), are widely used [1,5,9,17,20,21,22].

The use of PM signals can significantly increase the noise immunity of radar channels, and such systems include widely used PM-signals modulated by the M-sequence, codes, Frank, Barker and other sequences [1,9,14,15].

The AM of signals also has a wide practical application for radar systems [20,21,22,23], as well as for communication systems [24], for example, when generating ultra-wideband signals modulated in amplitude by Barker codes or an M-sequence [20,21,23], including with different values of the levels of elementary pulses of codes [22].

Barker codes are binary codes of finite lengths *N ≤ 13* composed of a symmetric alphabet {1, −1}. Until now, the question of whether it is possible to obtain perfect code sequences of longer than 13 characters has not been thoroughly investigated. It is known that there are no solutions for odd lengths with *N* values between 13 and 101. In addition, it is argued that there is little hope to find odd lengths exceeding 101 characters [25,26,27,28,29,30].

One of the ways to find solutions to the problem posed in this paper is to modify the known Barker codes up to a length of 13, as well as substantially longer M-sequences. To modify Barker codes and M-sequences with widely used values of the binary pair {1, −1}, it is proposed to switch to the values of the asymmetric pair {1, −*b*} and, in the general case, {*a*, −*b*}. Such a transition will enable us to mark the system of echo signals and, in contrast to the classical pair, will ensure the elimination of the ambiguity of their selection in a joint radar channel. It will also increase the number of positions by searching for several pairs that jointly yield uncorrelated code structures of the same length in the case of a multi-position SD AR.

The use of different pairs, such as {1, −*b*} and {*a*, −*b*}, makes it possible to expand the class of code sequences, extend the coding theory and methods of the digital complex processing of echo signals, as well as search for new code structures that have better correlation characteristics than the known ones. It also aids in the development of an appropriate technique for their search in the case of amplitude or phase modulation.

To achieve this, it is necessary to evaluate the characteristics of the ACF and perform a comparative analysis between the obtained new code sequences and the Barker codes of the considered lengths.

The search for the values of different pairs, such as {1, −*b*} and {*a*, −*b*}, for alternative code structures of lengths similar to Barker codes is reduced to tabular expressions obtained using the matrix method (see Table 1, Table 2, Table 3, Table 4 and Table 5).

**Figure 1:**
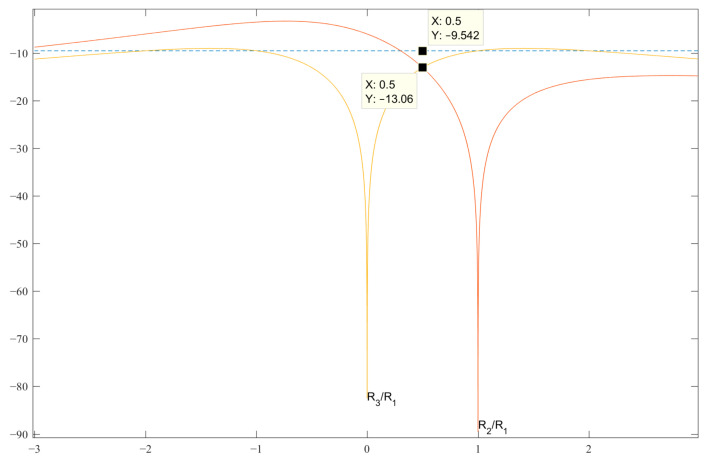
Graphs of the expressions SL 2 and 3 with normalized ACF for searching for *b* in the decibel measurement scale.

Figure 1 shows the graphs of the expressions SL 2 and 3 for finding the best value of b, at which the level of the side lobes of the normalized ACF to unity will be the minimum possible in the decibel measurement scale. The dotted line shows the level U of the SL values of the classical Barker codes of the length under consideration, and the SL expression (graphs) of the normalized ACF in Table 1 should not exceed this level U. Analyzing the graphs in Figure 1 and analytically solving the appropriate expressions for all of the SL—in this case, for SL 2 and 3—we obtain the code value *b* = 0.5 for the pair {1, −*b*}. This value is located at the intersection of the graphs of the expressions *R*_2_ and *R*_3_, which is shown in Figure 1. In this case, the optimal value of the maximum SL is *R*_2_ = *R*_3_= −13.06 dB.

In a similar way, expressions were determined to search for the pair {*a*, −*b*} (see Table 1), for which the SL values have a minimum level. The values of the pair {*a*, −*b*} are related through the expression *b* = 0.5*a*.

The expressions for searching for the modified Barker code structures of length *N* = 5 are given in Table 2.

**Figure 2:**
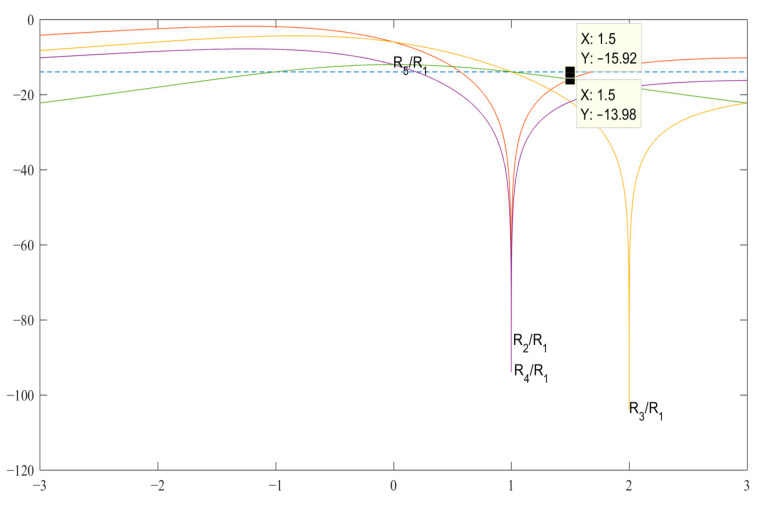
Graphs of expressions SL 2–5 with a normalized ACF for finding *b* in the decibel measurement scale.

As in the previous case with *N* = 3, analyzing the graphs of the expressions of the SL-s in Figure 2, as well as analytically solving the corresponding expressions for SL 2–5 (see Table 2), we obtain the value of the code *b =* 1.5 for the pair {1, −*b*}, with the level of maximum SL equal to *R*_2_ = *R*_5_ = −15.92 and *b =* 1.5*a* for the pair {*a*, −*b*}.

The expressions for searching for the modified Barker code structures of length *N* = 7 are given in Table 3.

**Figure 3:**
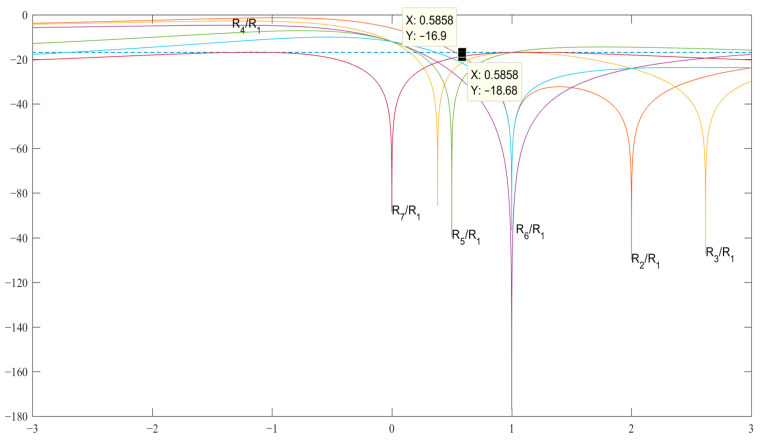
Graphs of expressions SL 2–7 with a normalized ACF for finding *b* in the decibel measurement scale.

In the case of *N* = 7 (see Table 3 and Figure 3) for the pair {1, −*b*}, the code value is obtained as $b=2-\sqrt{2}$, which allows us to obtain the SL level *R*_2_ = *R*_7_ = −18.68 dB and, accordingly, for the pair {*a*, −*b*} associated with the expression $b=\left(2-\sqrt{2}\right)a$. The expressions for searching for the modified Barker code structures of length *N* = 11 are given in Table 4.

**Figure 4:**
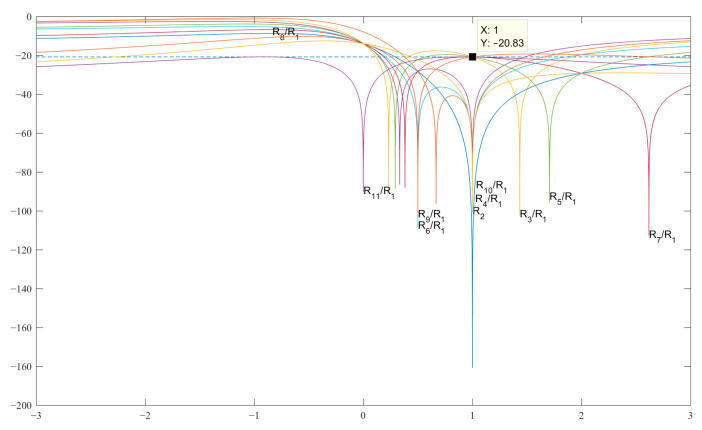
Graphs of the expressions SL 2–11 with a normalized ACF for finding *b* in the decibel measurement scale.

However, the analytically obtained values of the codes of the pair {1, *−b*} being substituted into the corresponding expressions in Table 4, showed SL levels (−19.11 dB) higher than those of classical Barker codes (see Figure 4). 

This means that there is no asymmetric code in terms of the SL level for the length *N* = 11, and the best code for amplitude modulation is the code with the value *b* = −1, while the level of all the SL is known to be equal to 1/11 or *−*20.83 in dB. The expressions for searching for the modified Barker code structures of length *N* = 13 are given in Table 5.

It should be noted that the logic for determining the expressions for calculating the levels of the ACF petals, given in Table 1, Table 2, Table 3, Table 4 and Table 5 are derived in a matrix way. To derive them, it is required to replace the elements of the code {1, *−*1} with {*a*, −*b*} in the original classical code of length *N*. Next, from the resulting modified code with new values of the elements {*a*, *−b*}, construct a cyclic matrix of dimension *N*×*N*, based on the shift of the code elements to the right. Further, the elements of the cyclic matrix are set to zero below the main diagonal. Then, the matrix obtained after zeroing is multiplied by the modified code in the form of a column with the values {*a*, −*b*}. As a result, expressions are derived for calculating each lobe of the ACF. The expressions for the pair {1, −*b*} are derived similarly.

**Figure 5:**
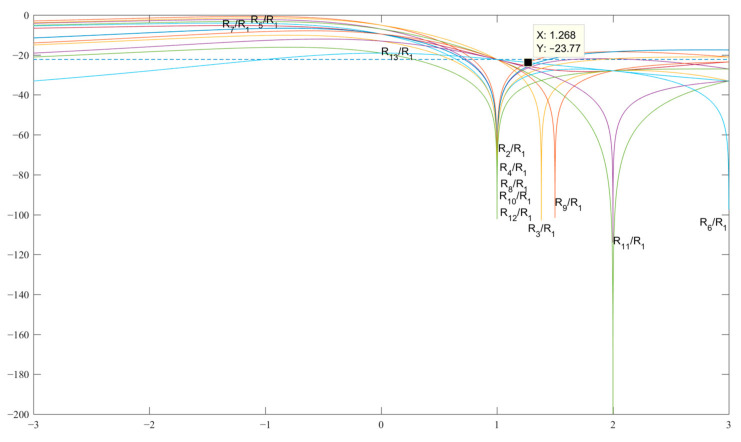
Graphs of expressions SL 2–13 with a normalized ACF for finding *b* in the decibel measurement scale.

Figure 5 shows the graphs of the expressions SL 2−13 as a result of the analysis, which found the value of b at which the SL level of the normalized ACF has the minimum possible value equal to *R*_2_ = *R*_13_ = −23.77 dB at $b=3-\sqrt{3}$ for the pair {1, −*b*} and, accordingly, the pair {*a*, −*b*} is related through the expression $b=\left(3-\sqrt{3}\right)a$.

At the same time, it should be noted that a pattern was noticed for the lengths of the codes *N* = 3, 5, 7 and 13, that the best values of the SL level are affected by SL expressions with numbers *R*_2_ and *R_N_*.

Thus, the new condition for the maximum amplitude of the SL in the case of replacement by {*a*, −*b*} or {1, −*b*} is determined by the expressions from Table 1, Table 2, Table 3, Table 4 and Table 5 with the numbers *R*_2_ and *R_N_*, after normalizing the ACF to unity; that is, after dividing them by *R*_1_.

It is also noteworthy that the code constructions of the lengths for *N* = 3, 7 and 11 differ by one element in the count of positive and negative symbols. Thus, a code with signs of elements (+ + −) of length *N* = 3 has two positive and one negative elements, a code (+ + + − − + −) of length *N* = 7 has four positive and three negative elements and a code (+ + + − − − + − − + −) of length *N* = 11 has five positive and six negative elements. The code structures of this type (having counts of negative and positive elements that differ by one) are determined by calculating the quadratic residues. In this case, the code length is determined by the expression *N*(*q*) *=* 4*q* − 1, where *q* is a natural number and when this is a prime number, *N* = 3, 7, 11, 19, 23, 31, 43 47, 59, 67, 71, 79, 83, 87, 91, 103, 107, 127, 131, 139, 151, 163, etc.

Code constructions of length *N* = 5 with signs (+ + + − +) and *N* = 13 (+ + + + + − − + + − + − +) structurally differ from lengths 3, 7 and 11. As you can see, the number of negative and positive elements for a code of length *N* = 5 are 1 and 4, respectively, and for a code of length *N* = 13, are 4 and 9, respectively. Consequently, the content of the code given by the number of negative elements corresponds to the square of a natural number — *q*^2^, and the number of positive elements, as it can be replaced, corresponds to the square of a number one greater than the previous one (*q +* 1)^2^. These code structures exist on lengths *N*(*q*) = *q*^2^ *+(q +* 1*)*^2^, *N* = 5, 13, 25, 41, 61, 85, 113, 145, 181, 221, 265, 313, 365, 421, 481, 545, 613, 685, etc. These code lengths are of less interest as the value of the code length grows faster; hence, there are large gaps among the possible lengths *N*, in contrast to the codes formed on the calculation of quadratic residues. The possibility of a smaller choice of code lengths during its generation is less attractive in the implementation of the SD AR as it may impose additional requirements when generating a probing signal.

Nevertheless, the results of the comparative analysis for evaluating the ACF characteristics for Barker code sequences and their modifications show that the SL level of the normalized ACF for a code sequence of length *N* = 7 is −18.68 dB, which is 1.78 dB lower than the SL level than for a similar Barker code. Of particular interest is the result where the SL level of the normalized ACF of length *N* = 3 exceeds the similar estimate for the Barker code by 3.52 dB. For a code sequence of length *N* = 11, the estimate of the SL level of the normalized ACF turned out to be worse than that of the classical representation of the Barker code by 1.72 dB. The results of a similar evaluation for codes showed that with a code length *N* = 5, its SL level is −15.92 dB lower than the similar evaluation of the Barker code equal to −13.98 dB, by 1.94 dB, and with *N* = 13 with values of −23.77 dB and −22.28 dB by 1.49 dB, respectively. Barker codes for lengths 2 and 4 are also known; however, asymmetric modified Barker code structures were not found for them as the expressions for their search—except for the classical symmetric binary solutions {1, −1}—do not exist in the presented approach and were therefore not considered separately.

Thus, the considered code constructions of lengths 3, 5, 7, 11 and 13 are modifications of Barker codes with an asymmetric alphabet. The analysis of the results of the computer experiments to compare the estimates of the autocorrelation characteristics of the widely used Barker codes and their modifications, similar in structure to code structures, showed the practical feasibility of this approach. This confirms the relevance of further research intended to find some new marked code structures with an asymmetric alphabet of pairs {1, −*b*} and {*a*, −*b*}, used to modulate probing signals in the SD AR.

Thus, as Barker codes exist up to length 13, the task is to search for modified code sequences that are similar in structure to codes of greater length and exceed their correlation properties, as well as having several options for marking within one length.

In this regard, it is advisable to develop a methodology for searching for the values of the elements of modified code structures for lengths *N* > 13, i.e., to move away from the classical approach when the alphabet of the code construction consists of ±1 and is symmetric. For the following structures similar to Barker codes, which are also the well-known M-sequences *N* = 2*^L^* − 1, *L* is an integer, *N* = 3, 7, 15, 31, 63, 127, 255, 511, 1023, etc. M-sequences are also proposed to be investigated for the expediency of their applicability as they have several implementations within the same length (several generating polynomials), which is required for the effective functioning of the SD; thus, they should be considered in more detail. In this case, the selected code structures should provide high compression characteristics [2,4,7] of broadband code structure systems, as well as a low degree of mutual influence during joint reception in the common channel of the SD of the small-sized AR.

## 3. Generation of M-Sequences

Among the code structures used to generate modulated signals, a special place in radar is occupied by code sequences of the maximum length or M-sequences. M-sequences belong to the category of binary linear recurrent sequences and represent a set of *N* periodically repeating binary symbols. As is known, the generation of binary values of the M-sequence is based on Galois fields [1,2,4,7]. When implemented in modern electronic systems, M-sequences are generated using shift registers with linear feedback.

In Table 6, some systems of polynomials are listed up to the degree *L* = 9, generating various code constructions of M-sequences. Tables of polynomials of higher orders are given in [1,4].

The M-sequences have the following advantages: a simple generation method, a low level of ACF side lobes and periodicity. These advantages are well studied and tested.

The study of their modifications with an asymmetric alphabet should be carried out separately for comparative analysis and the possibility of using them as code structures for the problems of detection, synchronization and error-correcting coding in the SD. This is because for each length *N* of the code, as mentioned earlier, there exist several generating polynomials of the same order, which is essential for the effective operation of the SD of the small-sized AR.

To demonstrate the cross-correlation properties of two different M-sequences, let us consider two polynomials of the same order—*g*_1_(*x*) = *x*^5^ + *x*^4^ + *x*^2^ + *x* + 1 and *g*_2_(*x*) = *x*^5^ + *x*^4^ + *x*^3^ + *x* + 1—each of which generates different M-sequences of length *N* = 31.

**Figure 6:**
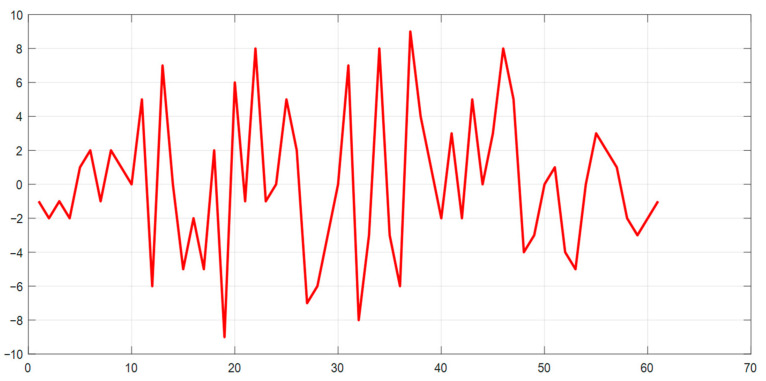
CCF of two M−sequences of the same length.

The correlation function of the above two M-sequences is shown in Figure 6. The CCF, as mentioned above, intrinsically has a uniform character at the level of the values of its petals, without pronounced peaks, which illustrates the possibility of using such code structures in the joint channel of the complex data flow of the SD AR.

## 4. Method for Searching for Modified M-Sequences

The method of searching for labeled code structures with the optimal value of *b*, taking into account the mutual correlation properties in the pair {1, −*b*} for M-sequences for the implementation of multi-position SD, is as follows.

**Figure 7:**
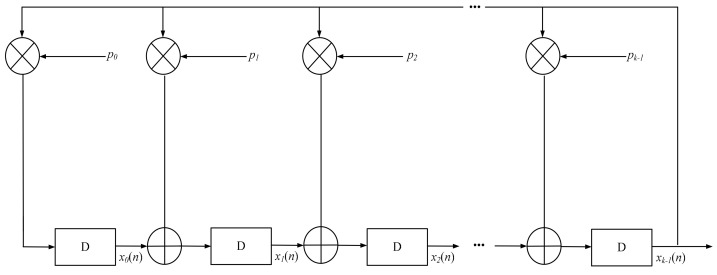
Diagram for generating the M-sequence.

(1) From Table 6, you need to choose several different polynomials, *g*_1_(*x*), *g*_2_(*x*), …, *g_n_*(*x*), of one order. The number of polynomials for the generation depends on the number of positions in the SD of the small-sized AR.
(2) Generate the required number of M-sequences based on the diagram (see Figure 7), *M*_1_, *M*_2_, …, *M_n_* sequences over polynomials *g*_1_(*x*), *g*_2_(*x*), …, *g_n_*(*x*), and bring their values to the symmetrical pair {1, −1}.

(3) Construct a normalized ACF for each of the M-sequences obtained at step 2.
(4) Determine the maximum modulo value of the SL of two ACFs obtained at step 3.
(5) In the generated code sequences at step 2, it is required to replace the code element from the value −1 to the value −*b*.
(6) Obtain the expressions for each lobe (main and side) of ACF depending on *b*.
(7) Obtain the expressions for each BL for the normalized ACF, thereby forming a system of SL expressions.
(8) Find the parameter *b* for which the SL value of the normalized ACF will be the smallest possible by solving the system of expressions obtained in step 7.
(9) Verify that the ACF SL values found in step 8 are lower than the maximum SL level determined in step 4.
(10) Make sure that the values of the CCF of the modified M-sequences *M*_1_, *M*_2_, ..., *M_n_* are uniformly distributed.

In this case, the marking of the code structures is associated with a different structure of polynomials for generating M−sequences of the same length. It is also associated, in some cases, with different found values of the −*b* parameter within the same code length. For example, generating M-sequences with the same polynomial will not give a marking of the code structure without using a technique for searching for the modification of negative elements of the −*b* code.

The method of searching for marked code structures for M-sequences with the pair {*a*, −*b*} is similar.

## 5. Computer Experiments on the Search for Labeled Code Structures based on Modifications of M-Sequences

In accordance with the methodology described in the previous section, two fifth-order polynomials were chosen to generate an M-sequence of length *N* = 31. The initial data of the first and second M-sequences, which are used to illustrate the nature of the ACF, as well as to generate their modifications, are as follows.

(1) Polynomial: *g*_1_(*x*) = *x*^5^ + *x*^2^ + 1 initial conditions for generation: [0 0 0 1 0].
(2) Polynomial: *g*_2_(*x*) = *x*^5^+ *x*^3^ + 1 initial conditions for generation: [0 0 0 0 1].

Next, it is required to find expressions that determine the level of ACF lobes for *g*_1_(*x*) and *g*_2_(*x*) with respect to *b* (see Table 7).

**Figure 8:**
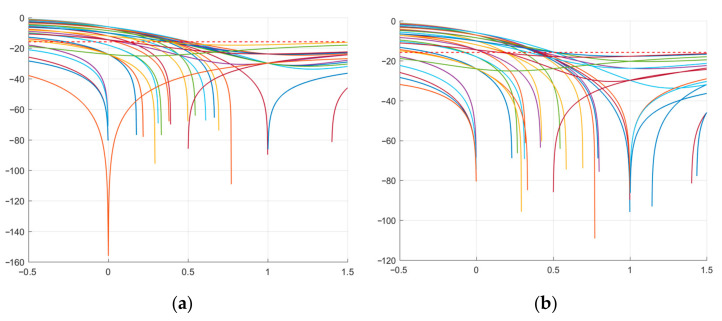
SL plots of the modified M-sequence: (**a**) SL expressions corresponding to *g*_1_(*x*); (**b**) SL expressions corresponding to *g*_2_(*x*).

Further, Figure 8 shows the graphs of the expressions in Table 7 relative to *b*.

**Figure 9:**
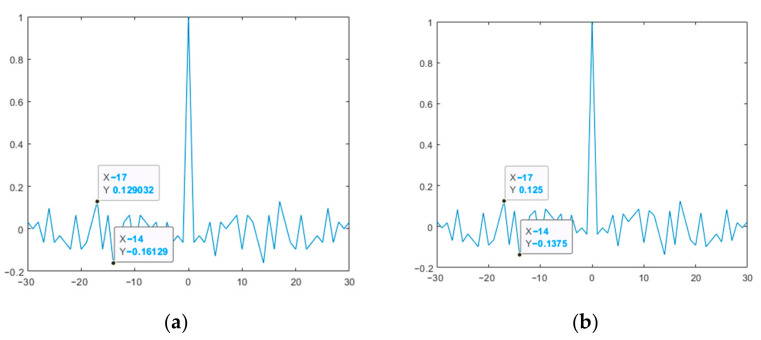
ACF of the studied M-sequences for the polynomial *g*_1_(*x*): (**a**) original; (**b**) modified.

**Figure 10:**
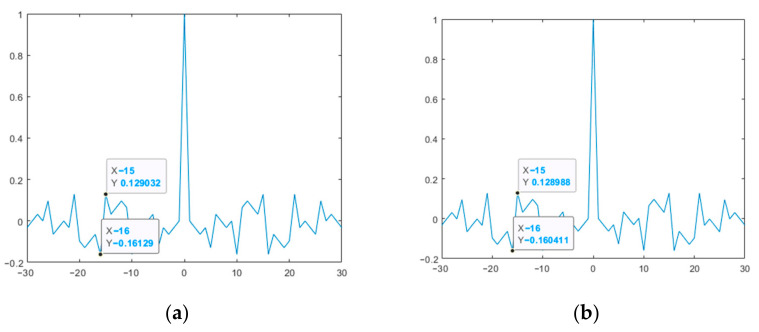
ACF of the studied M-sequences for the polynomial *g*_2_(*x*): (**a**) original; (**b**) modified.

Figure 9 and Figure 10 show the ACF plots of the original and modified M-sequences for the polynomials *g*_1_(*x*) and *g*_2_(*x*).

**Figure 11:**
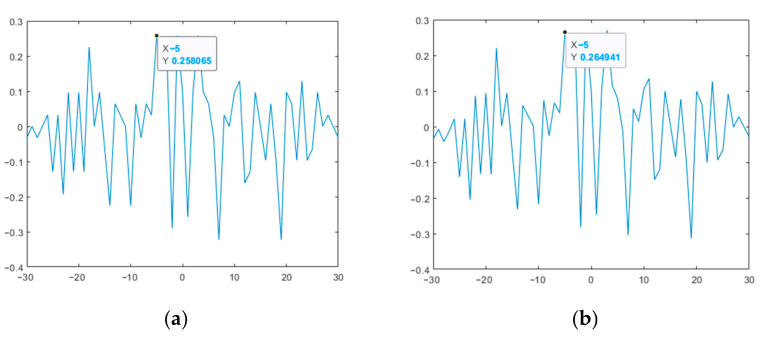
CCF of the two studied M-sequences: (**a**) original; (**b**) modified.

Figure 11 shows the CCF of the original and modified M-sequences.

Thus, the results presented in Figure 9 and Figure 10 show that the modified M-sequences are better than the original ones in terms of the ACF SL level. At the same time, the modification of the two M-sequences in this case does not affect the mutual correlation characteristics (see Figure 11). Similar studies were also given for other lengths of M-sequence systems. At the same time, it was verified that for the CCF inside such a system, it takes on a uniform character (UC) of the level of lobe values, as in the example in Figure 11.

Table 8 shows the results of this study; specifically, the selected systems of polynomials generating M-sequences of the same length for *N* > 13 are given, the values of *b* for their modification are determined, the estimate of the maximum SL level in dB and its difference from the SL level for the classical {1, −1} calculation of the normalized ACF, as well as the nature of the mutual influence of modified code structures within the system of generating sequences of the same length, are given.

From the analysis of the numerical results in Table 8, it follows that when applying the new technique described in Section 4, in which a new value of the negative element −*b* was searched for modifying M-sequences, it was possible to lower the level of SL compared to the classical representation. Therefore, for example, the largest decrease in the maximum SL was 2.6416 dB for the 5th degree polynomial *x*^5^ + *x*^4^ + *x*^3^ + *x*^2^ + 1, generating an M-sequence of length *N* = 31 with the value *b* = −0.6835, and the smallest SL decrease was 0.1959 dB for the 9th degree polynomial *x*^9^ *+ x*^8^ *+ x*^6^ *+ x*^5^ *+ x*^4^ *+ x*^3^ *+ x*^2^ *+ x +* 1, generating an M-sequence of length *N* = 511 with value *b* = −0.9186. The remaining similar numerical results are between these two indicated values of the SL levels.

Based on this, it should be concluded that it is expedient to use a new technique for searching for a new value of the negative element −*b* for modifying M-sequences.

Thus, a technique has been proposed that makes it possible to determine a new system of code structures for modulating probing signals while evaluating the autocorrelation and cross-correlation characteristics necessary for the implementation of mapping modes based on complex data processing in joint channels of the SD of the small-sized AR.

## 6. Conclusions

The actual task of searching for and investigating marked code structures for the echo signals of the SD of the small-sized AR is considered.

This paper presents the results of studies on evaluating the characteristics of new code sequences and performs comparative analysis with similar Barker codes for lengths 3, 5, 7, 11 and 13, often applicable in practice, as well as for modified M-sequences. The features of finding the values of the pairs {1, −*b*} and {*a*, −*b*} for the considered sequences are carried out.

The results obtained in the work and their analysis demonstrate the windows of opportunity associated with the asymmetry of the code and the weakening of the requirements for the ACF.

A technique has been developed, on the basis of which the search for new code structures necessary for marking complex probing signals in the implementation of the promising multi-positional SD of the small-sized AR has been carried out.

It is concluded that it is expedient to develop a method for searching for code values for modified M-sequences that can provide higher compression characteristics of systems of code structures, as well as a low degree of mutual influence during the joint reception and detection of an echo signal in the common channel of operation of the SD AR.

The result of this study is the basis for providing greater noise immunity in the selection of marked signals in common radio channels, as well as increasing the probability of their correct detection.

The results are intended to stimulate research in the field of revising signal generation and processing algorithms, increasing the reliability of detecting a useful signal in a complex interference environment (natural) in telecommunication and radar channels.

## Figures and Tables

**Table 1:** Expressions for searching for alternative Barker codes *N* = 3.

Lobe Number ACF	Search Expressions *b*	Expressions to Search for *a* and *b*
*R*_1_	*b*^2^ + 2	2*a*^2^ + *b*^2^
*R*_2_	−*b* + 1	*a*^2^ − *ab*
*R*_3_	−*b*	−*ab*

**Table 2:** Expressions for searching for modified Barker codes *N* = 5.

Lobe Number ACF	Search Expressions *b*	Expressions to Search for *a* and *b*
*R*_1_	*b*^2^ + 4	4*a*^2^ + *b*^2^
*R*_2_	−2*b* + 2	2*a*^2^ − 2*ab*
*R*_3_	−*b* + 2	2*a*^2^ − *ab*
*R*_4_	−*b* + 1	*a*^2^ − *ab*
*R*_5_	1	*a* ^2^

**Table 3:** Expressions for searching for modified Barker codes *N* = 7.

Lobe Number ACF	Search Expressions *b*	Expressions to Search for *a* and *b*
*R*_1_	3*b*^2^ + 4	4*a*^2^ + 3*b*^2^
*R*_2_	*b*^2^ − 3*b* + 2	2*a*^2^ − 3*ab* + *b*^2^
*R*_3_	*b*^2^ − 3*b* + 1	*a*^2^ − 3*ab* + *b*^2^
*R*_4_	(*b* − 1)^2^	(*a* − *b*)^2^
*R*_5_	−2*b* + 1	*a*^2^ − 2*ab*
*R*_6_	−*b* + 1	*a*^2^ − *ab*
*R*_7_	−*b*	−*ab*

**Table 4:** Expressions for searching for alternative Barker codes *N* = 11.

Lobe Number ACF	Search Expressions *b*	Expressions to Search for *a* and *b*
*R*_1_	6*b*^2^ + 5	5*a*^2^ + 6*b*^2^
*R*_2_	3*b*^2^ − 5*b* + 2	2*a*^2^ − 5*ab* + 3*b*^2^
*R*_3_	3*b*^2^ − 5*b* + 1	*a*^2^ − 5*ab* + 3*b*^2^
*R*_4_	3*b*^2^ − 4*b* + 1	*a*^2^ − 4*ab* + 3*b*^2^
*R*_5_	2*b*^2^ − 4*b* + 1	*a*^2^ − 4*ab* + 2*b*^2^
*R*_6_	2*b*^2^ − 3*b* + 1	*a*^2^ − 3*ab* + 2*b*^2^
*R*_7_	*b*^2^ − 3*b* + 1	*a*^2^ − 3*ab* + *b*^2^
*R*_8_	(*b* − 1)^2^	(*a* − *b*)^2^
*R*_9_	−2*b* + 1	*a*^2^ − 2*ab*
*R*_10_	−*b* + 1	*a*^2^ − *ab*
*R*_11_	−*b*	−*ab*

**Table 5:** Expressions for searching for alternative Barker codes *N* = 13.

Lobe Number ACF	Search Expressions *b*	Expressions to Search for *a* and *b*
*R*_1_	4*b*^2^ + 9	9*a*^2^ + 4*b*^2^
*R*_2_	*b*^2^ − 6*b* + 5	5*a*^2^ − 6*ab* + *b*^2^
*R*_3_	*b*^2^ − 5*b* + 5	5*a*^2^ − 5*ab* + *b*^2^
*R*_4_	*b*^2^ − 5*b* + 4	4*a*^2^ − 5*ab* + *b*^2^
*R*_5_	(*b* − 2)^2^	(2*a* − *b*)^2^
*R*_6_	*b*^2^ − 4*b* + 3	3*a*^2^ − 4*ab* + *b*^2^
*R*_7_	*b*^2^ − 3*b* + 3	3*a*^2^ − 3*ab* + *b*^2^
*R*_8_	−3*b* + 3	3*a*^2^ − 3*ab*
*R*_9_	−2*b* + 3	3*a*^2^ − 2*ab*
*R*_10_	−2*b* + 2	2*a*^2^ − 2*ab*
*R*_11_	−*b* + 2	2*a*^2^ − *ab*
*R*_12_	−*b* + 1	*a*^2^ − *ab*
*R*_13_	1	*a* ^2^

**Table 6:** Polynomial systems for generating M-sequences.

Degree of Polynomial *L*	The Length of the Generated Sequence *N* = 2*^L^* − 1	Type of Polynomials *g*(*x*) for Generating the M-Sequence
3	7	*x*^3^ + *x* + 1 *x*^3^ + *x*^2^ + 1
4	15	*x*^4^ + *x* + 1 *x*^4^ + *x*^3^ + 1
5	31	*x*^5^ + *x*^2^ + 1 *x*^5^ + *x*^3^ + 1 *x*^5^ + *x*^3^ + *x*^2^ + *x* + 1 *x*^5^ + *x*^4^+ *x*^3^ + *x* + 1 *x*^5^ + *x*^4^ + *x*^3^ + *x*^2^ + 1 *x*^5^ + *x*^4^ + *x*^2^ + *x* + 1
6	63	*x*^6^ + *x* + 1 *x*^6^ + *x*^4^ + *x*^3^ + *x* + 1 *x*^6^ + *x*^5^ + 1 *x*^6^ + *x*^5^ + *x*^2^ + *x*+ 1 *x*^6^ + *x*^5^ + *x*^3^ + *x*^2^+ 1 *x*^6^ + *x*^5^ + *x*^4^ + *x* + 1
7	127	*x*^7^ + *x* + 1 *x*^7^ + *x*^3^ + 1 *x*^7^ + *x*^3^ + *x*^2^ + *x* + 1 *x*^7^ + *x*^4^ + 1 *x*^7^ + *x*^4^ +*x*^3^ + *x*^2^ + 1 *x*^7^ + *x*^5^ + *x*^2^ + *x* + 1 *x*^7^ + *x*^5^ + *x*^3^ + *x* + 1 *x*^7^ + *x*^5^ + *x*^4^ +*x*^3^ + 1 *x*^7^ + *x*^5^ + *x*^4^ +*x*^3^+ *x*^2^ + *x* + 1 *x*^7^ + *x*^6^ + 1 *x*^7^ + *x*^6^ + *x*^3^ + *x* + 1 *x*^7^ + *x*^6^ + *x*^4^ + *x*^2^ + 1 *x*^7^ + *x*^6^ + *x*^4^ + *x* + 1 *x*^7^ + *x*^6^ + *x*^5^ + *x*^2^ + 1 *x*^7^ + *x*^6^ + *x*^5^ + *x*^3^ + *x*^2^ + *x* + 1 *x*^7^ + *x*^6^ + *x*^5^ + *x*^4^ + 1 *x*^7^ + *x*^6^ + *x*^5^ + *x*^4^ + *x*^2^ + *x* + 1 *x*^7^ + *x*^6^ + *x*^5^ + *x*^4^ + *x*^3^ + *x*^2^ + 1
8	255	*x*^8^ + *x*^4^ + *x*^3^ + *x*^2^ + 1 *x*^8^ + *x*^5^ + *x*^3^ + *x* + 1 *x*^8^ + *x*^5^ + *x*^3^ + *x*^2^ + 1 *x*^8^ + *x*^6^ + *x*^3^ + *x*^2^ + 1 *x*^8^ + *x*^6^ + *x*^5^ + *x* + 1 *x*^8^ + *x*^6^ + *x*^5^ + *x*^2^ + 1 *x*^8^ + *x*^6^ + *x*^5^ + *x*^3^ + 1 *x*^8^ + *x*^6^ + *x*^5^ + *x*^4^ + 1 *x*^8^ + *x*^6^ + *x*^4^ + *x*^3^ + *x*^2^ + *x* + 1 *x*^8^ + *x*^6^ + *x*^5^ + *x* + 1 *x*^8^ + *x*^7^ + *x*^2^ + *x* + 1 *x*^8^ + *x*^7^ + *x*^3^ + *x*^2^ +1 *x*^8^ + *x*^7^ + *x*^5^ + *x*^3^ +1 *x*^8^ + *x*^7^ + *x*^6^ + *x*^5^ + *x*^2^ + *x* +1 *x*^8^ + *x*^7^ + *x*^6^ + *x*^5^ + *x*^4^ + *x*^2^ +1 *x*^8^ + *x*^7^ + *x*^6^ + *x* +1
9	511	*x*^9^ + *x*^4^ + 1 *x*^9^ + *x*^4^ + *x*^3^ + *x* +1 *x*^9^ + *x*^5^ + 1 *x*^9^ + *x*^5^ + *x*^3^ + *x*^2^ + 1 *x*^9^ + *x*^5^ + *x*^4^ + *x* + 1 *x*^9^ + *x*^6^ + *x*^4^ + *x*^3^ + 1 *x*^9^ + *x*^7^ + *x*^2^ + *x* +1 *x*^9^ + *x*^7^ + *x*^5^ + *x* +1 *x*^9^ + *x*^7^ + *x*^5^ + *x*^2^ +1 *x*^9^ + *x*^7^ + *x*^6^ + *x*^4^ + 1 *x*^9^ + *x*^8^ + *x*^6^ + *x*^5^ + *x*^4^ + *x*^3^ + *x*^2^ +*x* +1 *x*^9^ + *x*^8^ + *x*^7^ + *x*^6^ + *x*^5^ + *x*^4^ + *x*^3^ + *x* +1

**Table 7:** Expressions for searching b corresponding to the polynomial *g_1_*(*x*) and *g_2_*(*x*).

Number Lobe ACF	Expressions for Finding *b* by *g*_1_(*x*) *L* = 5	Expressions for Finding *b* by *g*_2_(*x*) *L* = 5	Number Lobe ACF	Expressions for Finding *b* by *g*_1_(*x*) *L* = 5	Expressions for Finding *b* by *g*_2_(*x*) *L* = 5
1	15*b*^2^ + 16	15*b*^2^ + 16	17	3*b*^2^ − 9*b* + 3	2*b*^2^ − 10*b* + 3
2	6*b*^2^ − 16*b* + 8	7*b*^2^ − 15*b* + 8	18	5*b*^2^ − 5*b* + 4	2*b*^2^ − 8*b* + 4
3	6*b*^2^ − 15*b* + 8	6*b*^2^ − 15*b* + 8	19	4*b*^2^ − 6*b* + 3	2*b*^2^ − 8*b* + 3
4	5*b*^2^ − 15*b* + 8	5*b*^2^ − 15*b* + 8	20	3*b*^2^ − 7*b* + 2	2*b*^2^ − 8*b* + 2
5	6*b*^2^ − 13*b* + 8	5*b*^2^ − 14*b* + 8	21	2*b*^2^ − 7*b* + 2	2*b*^2^ − 7*b* + 2
6	4*b*^2^ − 15*b* + 7	4*b*^2^ − 15*b* + 7	22	3*b*^2^ − 4*b* + 3	4*b*^2^ − 3*b* + 3
7	5*b*^2^ − 12*b* + 8	5*b*^2^ − 12*b* + 8	23	2*b*^2^ − 6*b* + 1	3*b*^2^ − 5*b* + 1
8	5*b*^2^ − 12*b* + 7	5*b*^2^ − 12*b* + 7	24	2*b*^2^ − 5*b* + 1	3*b*^2^ − 4*b* + 1
9	5*b*^2^ − 11*b* + 7	4*b*^2^ − 12*b* + 7	25	2*b*^2^ − 4*b* + 1	2*b*^2^ − 4*b* + 1
10	5*b*^2^ − 10*b* + 7	4*b*^2^ − 11*b* + 7	26	2*b*^2^ − 4*b*	2*b*^2^ − 4*b*
11	4*b*^2^ − 12*b* + 5	3*b*^2^ − 13*b* + 5	27	3*b*^2^ − *b* + 1	3*b*^2^ − *b* + 1
12	5*b*^2^ − 9*b* + 6	5*b*^2^ − 9*b* + 6	28	*b*^2^ − 3*b*	2*b*^2^ − 2*b*
13	4*b*^2^ − 9*b* + 6	5*b*^2^ − 8*b* + 6	29	2*b*^2^ − *b*	2*b*^2^ − *b*
14	3*b*^2^ − 10*b* + 5	5*b*^2^ − 8*b* + 5	30	*b*^2^ − *b*	*b*^2^ − *b*
15	2*b*^2^ − 11*b* + 4	5*b*^2^ − 8*b* + 4	31	*b* ^2^	−*b*
16	4*b*^2^ − 7*b* + 5	5*b*^2^ − 6*b* + 5			

**Table 8:** The results of the selection of polynomials for the generation of modified M-sequences for the SD AR.

*N*	System Polynomials	The Value of *b* without Modification and with it	Level of SL in dB	The Difference from the SL Level at {1, −1}	The Nature of the CCF Values
15	*x*^4^ + *x* + 1	−1 −2	−11.4806 −13.0641	1.5835	UC
15	*x*^4^ + *x*^3^ + 1	−1 −0.6667	−11.4806 −13.9793	2.4987	UC
31	*x*^5^ + *x*^2^ + 1	−1 −0.7387	−14.2642 −16.0330	1.7688	UC
31	*x*^5^ + *x*^3^+ 1	−1 −0.7387	−15.8478 −17.3107	1.4629	UC
31	*x*^5^ + *x*^3^+ *x*^2^+ *x* + 1	−1 −1.5469	−14.2642 −15.2345	0.9703	UC
31	*x*^5^ + *x*^4^ + *x*^2^+ *x* + 1	−1 −0.7387	−15.8478 −17.6782	1.8304	UC
31	*x*^5^ + *x*^4^ + *x*^3^ + *x*^2^ + 1	−1 −0.6835	−14.2642 −16.9058	2.6416	UC
63	*x*^6^ + *x* + 1	−1 −1.3334	−20.4238 −20.7396	0.3158	UC
63	*x*^6^ + *x*^5^ + 1	−1 −0.7998	−17.9250 −19.1452	1.2202	UC
63	*x*^6^ + *x*^5^ + *x*^3^ + *x*^2^ + 1	−1 −1.3334	−19.0849 −19.2976	0.2127	UC
63	*x*^6^ + *x*^5^ + *x*^4^ + *x* + 1	−1 −1.3334	−17.9250 −18.7051	0.7801	UC
127	*x*^7^ + *x* + 1	−1 −1.2146	−21.2482 −21.7965	0.5483	UC
127	*x*^7^ + *x*^3^ + 1	−1 −0.8498	−21.2482 −22.1399	0.8917	UC
127	*x*^7^ + *x*^3^ + *x*^2^+ *x* + 1	−1 −1.2146	−21.2482 −21.4885	0.2403	UC
127	*x*^7^ + *x*^4^ + 1	−1 −0.8498	−20.4924 −21.0648	0.5724	UC
127	*x*^7^ + *x*^4^ + *x*^3^ + *x*^2^ + 1	−1 −1.2146	−20.4924 −21.1896	0.6972	UC
127	*x*^7^ + *x*^5^ + *x*^4^ + *x*^3^ + 1	−1 −0.8498	−21.2482 −22.0346	0.7864	UC
255	*x*^8^ + *x*^4^+ *x*^3^+ *x*^2^ + 1	−1 −0.8814	−23.5218 −24.2805	0.7587	UC
255	*x*^8^ + *x*^6^ + *x*^3^ + *x*^2^ + 1	−1 −0.8886	−24.0484 −24.4268	0.3784	UC
255	*x*^8^ + *x*^6^ + *x*^4^ + *x*^3^ + *x*^2^ + 1	−1 −0.8886	−23.5218 −24.1275	0.6057	UC
255	*x*^8^ + *x*^6^ + *x*^5^ + *x*^4^ + 1	−1 −0.8886	−24.0484 −24.6809	0.6325	UC
255	*x*^8^ + *x*^6^ + *x*^4^ + *x*^3^ + *x*^2^ + *x* + 1	−1 −0.8886	−23.5218 −24.1275	0.6057	UC
511	*x*^9^ + *x*^4^ + 1	−1 −0.9186	−27.7240 −28.0037	0.2797	UC
511	*x*^9^ + *x*^4^ + *x*^3^ + *x* + 1	−1 −1.0970	−26.9339 −27.2522	0.3184	UC
511	*x*^9^ + *x*^5^ + *x*^3^ + *x*^2^ + 1	−1 −0.8714	−26.9339 −27.5418	0.6080	UC
511	*x*^9^ + *x*^7^ + *x*^6^ + *x*^4^ + 1	−1 −0.8338	−26.9339 −27.6126	0.6787	UC
511	*x*^9^ + *x*^8^ + *x*^6^ + *x*^5^ + *x*^4^+ *x*^3^ + *x*^2^ + *x* +1	−1 −0.9186	−26.9339 −27.1297	0.1959	UC

## Data Availability

All data supporting the reported results are public.
